# Health and Social Care Integration: Insights from International Implementation Cases

**DOI:** 10.3390/jmahp13020028

**Published:** 2025-06-05

**Authors:** Ricardo Correia de Matos, Generosa do Nascimento, Adalberto Campos Fernandes, Cristiano Matos

**Affiliations:** 1 Department of Psychiatry and Mental Health, Unidade Local de Saúde da Região de Aveiro (ULSRA), EPE, 3810-164 Aveiro, Portugal; 2 Ordem dos Enfermeiros, 1600-848 Lisboa, Portugal; 3 Department of Human Resources and Organizational Behavior (IBS), ISCTE—University Institute of Lisbon, 1649-026 Lisboa, Portugal; generosa.nascimento@iscte-iul.pt; 4 National School of Public Health (ENSP), Universidade Nova de Lisboa, 1600-560 Lisboa, Portugal; adalberto.fernandes@ensp.unl.pt; 5 Atlântica School of Health, Atlantic University, 2730-036 Barcarena, Portugal; cristianom@uatla.pt; 6 Instituto de Saúde Ambiental (ISAMB), Faculdade de Medicina, Universidade de Lisboa, 1649-028 Lisboa, Portugal; 7 Laboratório Associado TERRA, Faculdade de Medicina, Universidade de Lisboa, 1649-028 Lisboa, Portugal; 8 Nursing Research, Innovation and Development Centre of Lisbon (CIDNUR), Nursing School of Lisbon (ESEL), 1600-096 Lisboa, Portugal

**Keywords:** integrated healthcare systems, intersectoral collaboration, patient-centered care, social determinants of health, health policy

## Abstract

The integration of health and social care is increasingly recognized as essential to address population ageing, the rise in chronic diseases, and persistent health inequities. Across Europe, diverse models have been developed to improve service coordination, resource efficiency, and person-centered care. This paper aims to explore international experiences in integrating health and social care, identify common strategies and challenges, and provide insights to inform policy development in countries where integration remains incipient, with a focus on Portugal. A qualitative comparative approach was employed. A systematic literature review was conducted across PUBMED, MEDLINE, and Google Scholar, including peer-reviewed articles, policy reports, and government documents. Thematic analysis was used to identify integration models, enablers, and barriers across different countries. Different models reveal that joint governance, pooled funding, strong community involvement, and digital innovation are key enablers of integration. However, common challenges persist, including fragmented governance, inconsistent implementation, and financial sustainability. In Portugal, structural separation between the health and social sectors continues to limit strategic alignment. Successful integration depends on political commitment, shared vision, and active stakeholder collaboration. European models offer adaptable lessons for Portugal and similar systems, especially regarding intersectoral coordination and preventive care. Integrating health and social care is vital for building resilient, equitable systems. Portugal must adopt a cohesive national strategy; strengthen local implementation; and embrace person-centered, sustainable solutions to ensure long-term impact. Integrating the health and social sectors is indispensable in navigating the ever-evolving healthcare landscape and promoting holistic well-being.

## 1. Introduction

The integration of the social and health sectors has become increasingly important, reflecting its growing relevance for society and public policy [1]. This integration refers to the collaborative effort to combine and align health and social sectors’ activities, resources, and expertise to improve a population’s overall health and well-being [2]. In Europe, integrating health and social care is a complex process that continues to evolve in response to political, social, and demographic changes [3,4]. In recent years, there has been a growing recognition of the need to foster collaboration and synergy between these health and social sectors, aiming to enhance healthcare quality and the overall well-being of the population [2,4]. Worldwide, healthcare faces significant challenges, notably population ageing and the escalating burden of chronic diseases [5,6]. By integrating the social and health sectors, it becomes possible to offer more efficient, patient-centered care and address clinical issues and the social determinants of health [7]. The growing recognition of the importance of this integration is mirrored in public policies and ongoing initiatives, which represent an opportunity to optimize existing resources and provide a more holistic and efficient approach to the population’s healthcare needs [8].

Some of the key aspects of this integration include (a) a holistic approach to healthcare, which goes beyond treating diseases and illnesses; (b) patient-centered care, with more personalized and patient-focused healthcare services; (c) efficiency and resource optimization, with a combination of resources from both sectors, reducing efforts, streamline services, and reducing the burden on the healthcare system; (d) preventive healthcare, addressing social determinants of health, such as poverty and education and housing, by tackling the root causes of illness before they develop; (e) equity and inclusivity, seeking to reduce health disparities and promote health equity; (f) community and public health, with integration extending beyond individual patient care to focus on the health of communities and the public as a whole; (g) and, finally, policy and collaboration between government agencies, healthcare providers, and other relevant stakeholders [9,10].

Europe has diverse healthcare systems, each combining public and private resources in different ways. Integrating health and social care is therefore seen as a valuable strategy to meet the population’s needs, while dealing with the challenges of a common health policy framework [11], including the complex needs of populations, particularly as they face common challenges such as ageing demographics, rising healthcare costs, and the increasing prevalence of chronic conditions [12]. Integration aims to overcome the traditional separation between healthcare and social services, offering more coordinated, person-centered support that reflects the broader social determinants of health [13].

This process involves creating collaborative frameworks between health institutions, local governments, and social care organizations, often supported by national or regional policy initiatives. These partnerships enable better coordination, more efficient resource allocation, and a more seamless experience for patients and families. However, the implementation of integration varies widely across countries, due to differences in healthcare delivery models, funding mechanisms, and governance structures [14].

Despite these challenges, the integration of health and social care is increasingly seen as essential to improving outcomes and ensuring system sustainability [1,2]. It reflects a broader commitment to patient-centered, holistic care that addresses both medical and social needs [15].

This perspective intends to present different integration examples in health and social care, analyze their implementation across various regions, and provide guidance for countries that are in the early stages of integration. Our objective is to present different integration models in health and social care, analyze their implementation across various regions, and provide guidance for countries that are in the early stages of integration. This paper also examines the case of Portugal, a country where integration is still emerging, offering insights into challenges and potential strategies for implementation.

## 2. Methods

The research strategy aimed to synthesize data from multiple sources to ensure a comprehensive and comparative analysis of integration models.

### Data Collection and Selection Criteria

A systematic literature search was conducted using PUBMED, MEDLINE, and Google Scholar to identify relevant peer-reviewed articles, government reports, and policy documents. The search included both original research articles and review studies, with no restrictions on publication date or geographical region, to capture the evolution of integration processes over time. Search terms included “integrated care model”, “health and social care”, “implementation”, “social and healthcare integration”, and related keywords. Additionally, reference lists of selected articles were manually reviewed to identify supplementary relevant sources. Data were selected based on their alignment with the study objectives, and prioritization was given to peer-reviewed journal articles and official government documents over grey literature to ensure academic rigor.

To systematically identify challenges and opportunities in health and social care integration, we conducted a comparative thematic analysis across different country models. This process involved reviewing policy documents to extract key implementation barriers and enablers, analyzing research findings on integration outcomes such as cost efficiency, patient-centered care, and intersectoral collaboration, and comparing integration frameworks to identify recurring challenges, including fragmentation, funding constraints, and governance complexity, as well as key opportunities, such as digital health adoption and policy harmonization.

## 3. General Overview of the Integration Process

The challenge of care integration requires the adequate measurement of policy implementation: the outputs and evidence of the integration have already been synthesized before [16]. Effective integration requires several key strategies, which include the development of interdisciplinary teams, improved coordination and communication between sectorial professionals, and strengthened support and training [17]. Enhancing access to resources and aligning clinical practices through shared guidelines and protocols also contributes to more cohesive service delivery [18]. Furthermore, successful integration depends on strong leadership and governance structures, capable of guiding collaboration across primary care, hospitals, and specialized services [19]. Equally important is the active involvement of patients and carers in the care process, reinforcing a patient-centered approach [20]. Lastly, the implementation of performance measurement systems is essential to monitor integration outcomes and ensure accountability, enabling continuous improvement based on real-world data [9,21,22]—Table 1.

Achieving financial sustainability is a critical component of successful integration, particularly for countries in the early stages of this process [23,24]. It requires strategic use of available resources; avoiding duplication; and promoting efficiency through coordinated service delivery and, where appropriate, consolidation [25]. Aligning financial incentives across sectors is essential to encourage collaboration and shared responsibility among healthcare and social care providers [26,27,28]. Emerging models such as pay-for-performance and bundled payments offer promising alternatives by focusing on the value and outcomes of care rather than service volume [29,30,31,32], which can support better coordination and shared accountability among providers [33,34]. In addition, performance-based budgeting, informed by data and analytics, can help identify inefficiencies and guide cost-saving decisions. Resource allocation can also benefit from economies of scale, such as joint procurement and streamlined administrative functions, and from strategic purchasing and contracting practices that contribute to the long-term sustainability of integrated systems [35,36].

### 3.1. International Integration Models: Comparative Perspectives

Across Europe, integration has emerged as a key strategy to improve service efficiency, population health outcomes, and the sustainability of healthcare delivery [4]. In other countries, such as the United States, integration efforts have also gained relevance but follow different paths due to distinct funding structures and policy environments [37]. Although countries differ in their approaches based on institutional structures, funding mechanisms, and governance models, many share the common goal of addressing the complex interplay between medical and social needs [9,38]. This section presents a comparative overview of integration efforts in different countries.

The integration of health and social care relies on strong policy coalitions that unite diverse stakeholders, including policymakers, healthcare providers, social workers, and community organizations. Sabatier’s Advocacy Coalition Framework (ACF) provides a valuable perspective on how these coalitions form, evolve, and shape policy decisions over time [39]. According to the author, policy actors with shared beliefs and objectives come together to form advocacy coalitions, working collectively to influence policy changes and implementation [39].

These coalitions typically emerge around key policy goals, such as improving patient-centered care, reducing system fragmentation, and optimizing resource allocation [40,41]. They can operate at multiple levels—national, regional, and local—with their effectiveness depending on factors such as political commitment, shared policy narratives, and institutional support. However, opposition from competing coalitions (e.g., those resistant to structural changes due to financial or ideological concerns) can hinder integration efforts, requiring negotiation, policy adaptation, and long-term advocacy strategies [42,43].

As different countries experiment with integration models, advocacy coalitions must continuously adapt their strategies based on real-world implementation outcomes. For example, successful integration in Scotland and the UK has demonstrated the importance of intersectoral collaboration and shared governance structures, which can serve as guiding principles for other nations. In contrast, in countries where integration efforts face resistance, ACF suggests that coalitions must mobilize resources, build strategic alliances, and actively engage policymakers to overcome barriers and drive sustainable change.

#### 3.1.1. United Kingdom and Scotland

The United Kingdom has made considerable progress in health and social care integration, particularly through the National Health Service (NHS) in England [38,44] and the model implemented in Scotland [24]. Scotland has institutionalized integration through Health and Social Care Partnerships (HSCPs), created under the Public Bodies (Joint Working) (Scotland) Act 2014 [21,45]. These partnerships align health boards and local authorities, sharing governance and financial responsibilities to deliver unified care [24].

The Scottish model emphasizes person-centered care, joint budgeting, and a focus on community-based preventive services. Successes include improved care continuity, reduced hospital admissions, and increased user satisfaction [24]. However, challenges remain in regional implementation disparities, workforce integration, and sustaining long-term funding alignment [16]. Despite these, Scotland is often cited as a leading European model of health and social care integration.

#### 3.1.2. Sweden

Sweden has developed innovative, patient-centered integration models such as the Norrtaelje Model and the Esther Project [46]. The Norrtaelje initiative combines healthcare and municipal social services under a single governance structure and budget, facilitating coordinated service delivery for elderly patients and those with chronic conditions [47]. The Esther Project uses patient narratives to redesign care processes, ensuring that services are aligned with individual experiences and needs [48,49]. These initiatives demonstrate how collaborative governance and localized integration can reduce service fragmentation and improve outcomes. Sweden’s emphasis on shared decision-making and community involvement sets a valuable precedent, although implementation can be resource-intensive and dependent on strong local leadership and administrative capacity [46,50].

#### 3.1.3. Germany

Germany’s health and social care integration is shaped by a federal and insurance-based system, resulting in significant regional variation. One of the most advanced initiatives is the Gesundes Kinzigtal model, a population-based, value-oriented care approach in the Black Forest region [51,52]. It brings together physicians, insurers, and community organizations to promote preventive care and manage chronic diseases more effectively [51,53]. The model has demonstrated improvements in health outcomes and cost savings, particularly through early intervention and integrated care pathways. However, national scalability is limited by decentralized governance and the structural separation of insurance funds. Nonetheless, Germany illustrates how locally tailored, insurer-provider partnerships can drive integration within a pluralistic system [51,52].

#### 3.1.4. Czech Republic

The Czech Republic has achieved universal health coverage with relatively low public health expenditure, yet its integration of health and social services remains underdeveloped [54]. The system is primarily hospital-based, with limited coordination between medical and social care services. Family members provide the majority of elder care, with over 80% of older adults relying on informal caregiving. Integration challenges include insufficient health IT infrastructure, fragmented governance, and inadequate home-based care services. However, there is growing policy interest in transitioning towards more community-oriented care and enhancing support for family caregivers, marking the beginning of a more integrated approach [55].

#### 3.1.5. Denmark

Denmark’s health and social care integration is widely regarded as one of the most comprehensive in Europe [3,56]. Services are coordinated at the municipal level, allowing for seamless transitions between hospital, primary, and social care [56]. The country emphasizes preventive health measures, digital infrastructure, and community-based interventions [57]. Health and social services are universally available based on need, regardless of age or income, and are fully financed through general taxation. For older adults, a broad and varied set of integrated services is provided, including home help, home nursing, rehabilitation, and nursing homes, ensuring that care is available without institutionalization, except in the most complex cases. In fact, Denmark has very few institutions outside the hospital system for the frailest elderly, as municipalities are responsible for meeting their needs through community-based, integrated care. This model is underpinned by the principle of self-care, which views individuals as autonomous and capable of making decisions about their own health and life. This emphasis on independence aligns with Denmark’s broader ageing policy, which supports dignity, autonomy, and active participation in society [57].

#### 3.1.6. Italy

Italy demonstrates a unique approach to integration through its mental health reform in the city of Trieste, widely recognized as a pioneering model of community-based care [58]. Since the 1970s, Trieste has moved away from institutional psychiatry by closing large mental hospitals and replacing them with open-door services, multidisciplinary teams, and strong community support [58,59]. This model prioritizes patient autonomy and social inclusion, offering personalized care in community settings. Despite ongoing financial and political constraints, Trieste’s system continues to deliver effective, rights-based mental healthcare [58,59]. Italy’s experience highlights the role of local leadership, civic engagement, and human rights principles in driving integration, particularly in decentralized systems [58,59].

#### 3.1.7. The Netherlands

The Netherlands combines universal social health insurance with innovative, decentralized care delivery models [60]. Buurtzorg Nederland, a nurse-led, self-managed home care organization, exemplifies effective integration at the community level [61]. The model has achieved high levels of patient satisfaction, workforce engagement, and cost-efficiency [61]. Efforts are ongoing to improve interoperability of digital systems and cross-sector workforce coordination, which are critical for further integration. The Dutch experience illustrates how patient-centered innovation and decentralized governance can coalesce into scalable, sustainable integration models.

#### 3.1.8. United States of America (USA)

The USA healthcare system is largely market-driven, supported by a mix of public and private funding, which presents challenges for achieving effective integration between health and social care. Historically, integration efforts have been limited, with a growing focus in recent years on addressing social determinants of health. Notably, the Medicaid program, established in 1965 and covering around 20% of the population, has become a key vehicle for integration, operating through a joint federal–state funding model with localized administration [37,62]. The Affordable Care Act enabled several states to expand Medicaid, extending access to low-income populations and reinforcing links between social needs and health coverage [63]. Innovative approaches, such as Social Impact Bonds, have also supported integration efforts by funding preventive interventions. Despite this progress, significant barriers remain. The U.S. healthcare system is highly fragmented, and political and ideological divisions continue to hinder cohesive, nationwide integration policies [64].

The comparison between countries reveals fundamental differences in integration models, primarily driven by healthcare financing structures and policy governance [65]—Table 2. While European countries, particularly Scotland and the UK, have embraced publicly funded, government-led integration strategies, the USA remains largely market-driven, with fragmented coordination between healthcare and social services. Scotland’s model, with its centralized governance and shared funding, provides a strong contrast to the USA approach, where financial incentives are often misaligned, and integration depends on state-level initiatives and Medicaid expansion efforts. This comparison underscores the impact of governance structures, funding models, and policy commitment in shaping successful integration efforts.

The UK boasts a publicly funded NHS that offers all-encompassing healthcare services and has a longstanding tradition of integrating health and social services [66]. The NHS is a publicly funded system that provides most healthcare for free. Some services, like emergency treatment, family planning, and care for certain infectious diseases, are free for everyone. However, other services, like dental care, prescriptions, or non-urgent treatments, may have charges, especially for people who do not meet certain exemption rules. Common challenges include patients with disabilities; the closure of public hospitals dedicated to those with physical, mental, and developmental disabilities; escalating costs related to acute and long-term care; the trend toward shorter acute-care stays with an emphasis on community-based care; and the overarching fiscal pressures experienced at both the national and local levels [67].

In a seminal paper, Leutz proffers pivotal recommendations for integrating the social and health sectors [68] regarding the importance of involving service users, carers, and community service providers in planning and oversight processes and the necessity of developing systems that seamlessly integrate, coordinate, and connect services for individuals with disabilities, whilst also clarifying the demarcation between medical and non-medical systems [68]. The UK’s integration of health and social care, led by the NHS and local authorities, reflects strong policy commitment and coordination efforts but faces challenges related to funding pressures and regional disparities in implementation.

Scotland has introduced a healthcare integration model that divides responsibilities between health boards and local authorities—a strategy that has shown notable success, as highlighted in a comprehensive study by Bruce and Parry [69]. The core administrative entities in this system are Health and Social Care Partnerships (HSCPs), collaborative arrangements forged between local authorities and health boards, and institutionalized manifestation sanctioned through the Public Bodies (Joint Working) (Scotland) Act 2014 [70]. These HSCPs are tasked with ensuring that healthcare and social services are dispensed in a manner that is not only synchronized but, more crucially, deeply attuned to the singular needs and aspirations of the individuals within their purview [69].

The Scottish model of integration offers several notable strengths but also presents challenges that highlight the complexities of implementing such frameworks [21]. One of its primary advantages is its comprehensive governance structure, with Health and Social Care Partnerships (HSCPs) ensuring close collaboration between health services and local authorities. Additionally, joint budgeting mechanisms have allowed for more efficient resource allocation, reducing duplication and enhancing patient-centered care. Scotland’s focus on preventive care and early intervention has also contributed to improved long-term health outcomes [21]. However, despite these strengths, integration in Scotland has faced challenges related to funding sustainability, workforce coordination, and variations in implementation across regions. Some areas struggle with operational inconsistencies, where differences in local governance lead to variability in service effectiveness. Additionally, the integration process requires continuous policy adaptation, as shifting demographic trends and evolving healthcare demands create new pressures on the system. While Scotland’s approach remains a leading example of successful integration, ongoing efforts are needed to streamline coordination, address financial constraints, and ensure consistency in service delivery across all regions [21].

Scotland’s integration model is grounded in person-centered care, community-based support, and early intervention, aiming to improve quality of life and reduce hospital admissions [71]. It emphasizes preventive strategies and addressing social determinants of health to reduce inequalities. Strong governance, clear health and wellbeing outcomes, community involvement, and the use of digital tools support implementation. Funding is pooled across sectors to ensure flexible and efficient resource use, prioritizing individual needs over institutional boundaries [70,72].

### 3.2. Integration of Health and Social Sectors in Portugal: A Complex and Vital Endeavor

As the demographic and chronic disease burden grows, the need for integrated care becomes increasingly pressing, and the European models discussed provide both inspiration and guidance for Portugal’s next steps.

Portugal operates a National Health Service (SNS) that provides universal coverage, yet health and social care remain structurally separated [73]. Healthcare is centrally governed by the Ministry of Health, whereas social services are managed by municipalities and third-sector organizations—non-profit private entities, mostly associations, including religious institutions and foundations. This division limits strategic alignment and coordination between health and social care sectors. Recent developments include Local Health Units (ULS), which attempt to combine healthcare and some social functions under a shared structure [74], and the Integrated Continuous Care Network (RNCCI), designed to offer long-term, rehabilitative, and palliative care [75].

One of the distinct features of the Portuguese system is its high degree of centralization in healthcare policy alongside strong municipal autonomy in social services, which often leads to mismatched priorities and a lack of shared planning. Furthermore, the strong presence of third-sector organizations, including religious and philanthropic institutions, plays a significant role in long-term and social care, which can create both opportunities for innovation and challenges in accountability.

Portugal could benefit from the experience of successful European models, such as Scotland’s joint governance and budgeting frameworks, Sweden’s patient-centered coordination strategies, and Germany’s efforts toward regional integration. Despite some progress, Portugal continues to struggle with fragmented governance, funding asymmetries, and regional disparities in service provision. To address these issues, Portugal should consider piloting regional pooled budgeting mechanisms between health and social sectors, particularly in areas with ageing populations and high chronic disease burden. These pilots could be supported by integrated digital case management systems that facilitate data sharing and coordinated care planning across services.

Strengthening intersectoral collaboration, adopting digital health solutions, and developing a unified funding strategy could significantly advance the integration process and promote more patient-centered care. To achieve sustainable progress, a more cohesive and strategic national approach is essential for the full integration of health and social care. Moreover, a national policy framework that incentivizes joint commissioning and formal intersectoral agreements between health institutions and municipalities could strengthen accountability and ensure alignment of goals and resources.

This integration is increasingly recognized as a necessary response to the challenges affecting system sustainability, population well-being, and the broader social determinants of health [76]. However, the process is inherently complex, shaped by intersecting political, economic, demographic, and social factors. National and regional policies aim to support these partnerships, encouraging coordinated action, more efficient resource allocation, and improved service delivery centered on the needs of individuals [77].

Demographic trends such as population ageing, rising healthcare costs, and the increasing burden of chronic diseases reinforce the urgency of integration. Regional and local autonomy adds further complexity, often hindering strategic alignment and effective coordination across sectors [78].

Building on international examples, Portugal could establish regional “integration zones” with dedicated governance teams, shared performance indicators, and budget flexibility to test different coordination mechanisms in real-world settings. To overcome these structural and systemic barriers, Portugal must commit to a long-term vision for integration that includes clear governance frameworks, robust investment in community-based services, and active involvement of all stakeholders—from national authorities to local providers and civil society. Only through a coordinated, person-centered, and sustainable approach can Portugal ensure equitable access, improve outcomes, and build a resilient health and social care system fit for future challenges.

From a theoretical standpoint, the ACF offers useful insights into the Portuguese case. Although there is growing awareness of the need to integrate health and social care, the lack of a cohesive advocacy coalition—bringing together actors from the Ministry of Health, municipalities, third-sector organizations, and civil society—has hindered the emergence of a unified vision. Stakeholders often operate with divergent policy beliefs and priorities, shaped by sectoral silos and institutional inertia. Health sector actors tend to follow a centralized, medicalized logic, while social sector entities emphasize local autonomy and social support. These differing perspectives limit the development of shared narratives and coordinated action. Furthermore, some resistance persists among stakeholders concerned about the redistribution of resources or loss of institutional autonomy.

## 4. Conclusions

Integrating health and social sectors has emerged as a paramount strategy in the modern healthcare landscape, particularly in the face of demographic change, rising chronic disease burdens, and increasing service demands. This study has explored the multifaceted dynamics of integration across various countries, highlighting the profound interplay between medical care and social determinants of health. International models—such as those from Scotland, Sweden, and Germany—demonstrate that collaborative governance, joint funding, and person-centered approaches can enhance service coordination, reduce inefficiencies, and improve population health outcomes.

In Portugal, despite some promising initiatives like Local Health Units (ULS) and the Integrated Continuous Care Network (RNCCI), structural fragmentation, funding asymmetries, and lack of strategic alignment still hinder full integration. The persistence of these barriers underscores the need for a cohesive national strategy that prioritizes intersectoral collaboration, invests in community-based services, and promotes digital innovation.

While the challenges of fragmentation, funding constraints, and political complexity persist, the potential benefits of integration are clear: improved equity, better health outcomes, the more efficient use of resources, and services that are truly centered on people’s needs. Achieving this vision requires unwavering collaboration between all stakeholders—from central government to local providers, civil society, and communities—and a firm commitment to addressing the broader determinants of health.

## Figures and Tables

**Table 1:** Measures for assessment of health and social care integration.

Measures	Outcomes
Structural	Improved collaboration between social care and healthcare professionals Improved staff perceptions Improved support and training for care home staff Improved access to resources Improved impact in specific clinical care settings
Processes	Improved quality of care standards (inconclusive) Improved prescribing rates (limited) Improved self-management in older people with multiple chronic conditions Improved patient satisfaction Improved staff working experience (inconclusive) Improved integration and coordination of services
System outcomes	Decreased hospitalization Decreased length of stay (inconclusive) Decreased unscheduled admissions (inconclusive) Decreased admissions and readmissions (inconclusive) Increased number of clinician contacts (inconclusive)Improved access and availability of services Decreased waiting times Reduced costs (inconclusive) Decreased time in emergency departments Improved health equity
Health outcomes	Improved clinical outcomes Improved quality of care Decreased mortality (inconclusive) Improved quality of life
Patient and carer reported outcomes	Improved patient satisfaction and wellbeing Improved physical health Improved psychological and social wellbeing Improved perceptions among carers and families

Adapted from Matos et al. [16].

**Table 2:** Comparative table of health and social care integration models.

Country	Integration Model	Key Features	Challenges
United Kingdom and Scotland	Health and Social Care Partnerships (HSCPs) with joint governance and budgeting	Person-centered care, community-based services, and joint budgeting	Regional disparities, funding alignment, and workforce integration
Sweden	Norrtaelje Model and Esther Project—joint structures and patient-centered design	Shared decision-making, local leadership, and patient narratives in design	Resource-intensive, depends on strong local leadership
Germany	Gesundes Kinzigtal—insurer-provider partnerships focused on value-based care	Early intervention, regional tailoring, and outcome-based funding	Scalability due to decentralization and insurer fragmentation
Czech Republic	Mainly hospital-based system with limited integration; informal elder care	Low public spending, reliance on family caregivers, and policy interest in reform	Fragmented governance, lack of home-based services and IT systems
Denmark	Municipal-level coordination; emphasis on preventive and home-based care	Universal access, local delivery, and emphasis on autonomy and self-care	Maintaining equity and consistency across municipalities
Italy	Trieste’s community-based mental health model with strong civic engagement	Open-door psychiatric care, multidisciplinary teams, and rights-based approach	Budgetary and political constraints
The Netherlands	Buurtzorg—nurse-led, self-managed home care, and decentralized innovation	High satisfaction and efficiency, strong community presence, and digital efforts	Ensuring digital interoperability and workforce coordination
USA	Medicaid expansion and Social Impact Bonds; limited by system fragmentation	Public-private mix, focus on social determinants, and political/structural barriers	System fragmentation, political resistance to nationwide policies

## Data Availability

No new data were created or analyzed in this study.
